# Development of Nationwide Road Quality Map: Remote Sensing Meets Field Sensing

**DOI:** 10.3390/s21062251

**Published:** 2021-03-23

**Authors:** Sadra Karimzadeh, Masashi Matsuoka

**Affiliations:** 1 Department of Remote Sensing and GIS, University of Tabriz, Tabriz 5166616471, Iran; 2 Institute of Environment, University of Tabriz, Tabriz 5166616471, Iran; 3 Department of Architecture and Building Engineering, Tokyo Institute of Technology, 4259-G3-2 Nagatsuta, Midori-ku, Yokohama 226-8502, Japan; matsuoka.m.ab@m.titech.ac.jp

**Keywords:** international roughness index, road quality, remote sensing, Sentinel-2

## Abstract

In this study, we measured the in situ international roughness index (IRI) for first-degree roads spanning more than 1300 km in East Azerbaijan Province, Iran, using a quarter car (QC). Since road quality mapping with in situ measurements is a costly and time-consuming task, we also developed new equations for constructing a road quality proxy map (RQPM) using discriminant analysis and multispectral information from high-resolution Sentinel-2 images, which we calibrated using the in situ data on the basis of geographic information system (GIS) data. The developed equations using optimum index factor (OIF) and norm R provide a valuable tool for creating proxy maps and mitigating hazards at the network scale, not only for primary roads but also for secondary roads, and for reducing the costs of road quality monitoring. The overall accuracy and kappa coefficient of the norm R equation for road classification in East Azerbaijan province are 65.0% and 0.59, respectively.

## 1. Introduction

Since ancient times, roads have played an important role in connecting different geographic locations to each other. Currently, in the face of rapid urbanization and industrialization, the role of roads is even more important because they not only allow people to travel from one location to another but also contribute to the economic growth of nations [1]. If the surface evenness, rideability, quality, skid resistance, or other influential factors are below the defined standards, a road can be fixed or require maintenance [2]. Poorly maintained roads can seriously affect economy, fuel consumption, and mobility, while further increasing driving costs, air pollution, and maintenance/operating costs for both roads and vehicles. From this economic perspective, road quality mapping is an important task for enabling safe, smooth, and efficient travel. Roads with better quality also enable farmers to work in more distant fields and distribute their products more efficiently.

For the general population, “road quality” denotes the level of roughness of the road, along with other parameters, such as number of lanes, potholes, signage and layout. Several roughness indices have been developed to date. The international roughness index (IRI), introduced in the early 1980s, is one such index that is basically obtained from the measured longitudinal road profile [3,4,5]. The IRI is a measure of the displacement in the vertical direction per horizontal distance traveled on the road. In other words, the IRI, which is expressed in units of meters/kilometer or inches/mile, represents a roughness quality that affects vehicle performance on the road. Although ground-based methods for measuring the IRI are of most interest, remote sensing methods are emerging as an alternative for acquiring in situ IRI measurements. Remote sensing techniques are capable of measuring various types of information without physical contact (only through electromagnetic waves). Therefore, remote sensing measurements can be recorded using various platforms, such as unmanned aerial vehicles (UAVs), airplanes, and satellites, from which many infrastructure changes can be detected and monitored. A few examples of such remote sensing applications to infrastructure monitoring include observing infrastructure damage after natural and man-made disasters, planning long-term road consolidation, and detecting dam instabilities [6,7,8,9,10,11,12,13,14,15].

**Figure 1:**
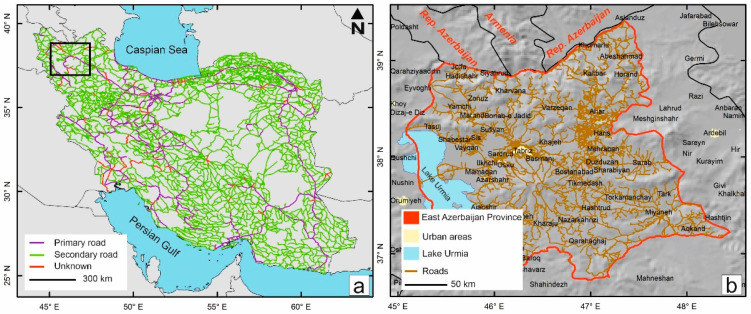
(**a**) Road network map of Iran. The black box denotes the study area. (**b**) Road network of East Azerbaijan Province, Iran, the study area.

Figure 1a shows the total length (approximately 214,000 km) of the road network in Iran, including primary (freeway and highway), secondary (major and local), and rural roads. East Azerbaijan Province, one of the 31 provinces in Iran with a road network extending up to approximately 14,000 km, plays an important role in national road networking because it connects Iran to Armenia and the Republic of Azerbaijan. In this work, we measured the IRI on major roads throughout East Azerbaijan Province of Iran using a smartphone equipped with a motion-sensitive “BumpRecorder” and “Physics Toolbox” applications and a quarter car (QC). The BumpRecorder application did not require any specific calibration, since its accuracy was tested several times before the commercial operation [16]. These measurements were acquired by driving back and forth only between the capital of the province, Tabriz, and the counties shown in yellow in Figure 1b. The road map of East Azerbaijan Province (Figure 1b) is produced from the OpenStreetMap (OSM) database. Using in situ data as training samples, combined with remote sensing data (featuring a broader coverage of the desired region), incomplete information about different objects or features can be completed. Accordingly, we regressed independent variables obtained from a Sentinel-2 dataset on the dependent variables (field data) to analyze the road network of the whole country.

## 2. Data and Methodology

### 2.1. In Situ Measurements

**Figure 2:**
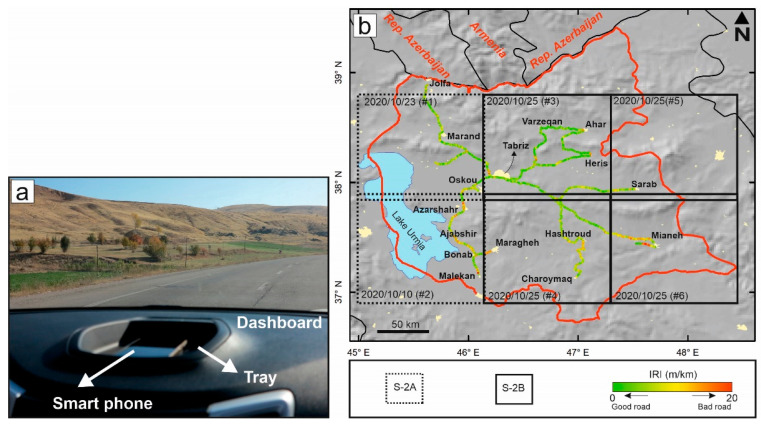
(**a**) Setup for conducting international roughness index (IRI) measurements in East Azerbaijan Province using a quarter car (QC) and a smartphone. (**b**) The resulting back-and-forth in situ IRI measurements in East Azerbaijan Province of Iran from Tabriz along the primary roads leading to the other counties. Black boxes show the areas covered by Sentinel-2 images of the province. Dashed boxes indicate the images acquired from Sentinel-2A, and solid black boxes indicate the images acquired from Sentinel-2B.

Engineers working in the transportation sector typically use a “one-wheel” vehicle to acquire traditional field measurements of road quality. These vehicles are suitable for precise IRI measurements because the roughness measurements are not affected by the suspension system [16,17]. In contrast, the QC method was not originally popular among engineers because the QC has a suspension system. Therefore, due to this suspension system, the recorded acceleration might be affected by the car type and driving speed. The roughness measurements recorded with a QC are also not typically stable, and it is necessary to perform a driving calibration before taking measurements. However, the extensive use of smartphones has marked a paradigm shift for road quality mapping. In this study, we used the BumpRecorder application developed by BumpRecorder Co., Ltd., to measure the acceleration and longitudinal profile of the road and refine the results using vibration frequency analysis. The use of this application therefore eliminates the need for a driving calibration step. Although the type of car does not seriously affect the measurements, we used only one car for the entire project. As shown in Figure 2a, we mounted a smartphone (Huawei Y7) equipped with an accelerometer and a global positioning system (GPS) sensor on the dashboard tray of a long chassis car (CHANGAN-CS35) to acquire IRI measurements on the roads (e.g., highways and major roads) throughout East Azerbaijan Province.

Unfortunately, even with a QC, the measurement of major roads is time-consuming because there are often several lanes and driving on the same road several times to record the quality of each lane requires additional labor and time. To reduce the time required to perform these ground-based in situ measurements, we drove on only one lane on each road, with priority given to the lane with the worst road quality. Due to the cold winters and the mountainous topography of the study area, the measurements were gathered from 13 October 2020 to 30 October 2020 to avoid dangerous driving conditions. We took measurements on major roads spanning more than 1300 km throughout East Azerbaijan Province, Iran, with an average speed of 80 km/h and a sampling frequency of 200 Hz. According to Karimzadeh and Matsuoka (2020), the accuracy of the QC method is acceptable for major roads, but on urban streets, tall buildings and traffic jams may reduce the quality of simultaneous location and acceleration measurements [4]. Figure 2b shows the IRI field measurements every 200 m, in which green, yellow-orange, and red colors represent smooth roads, intermediately rough roads, and the roughest roads, respectively.

**Figure 3:**
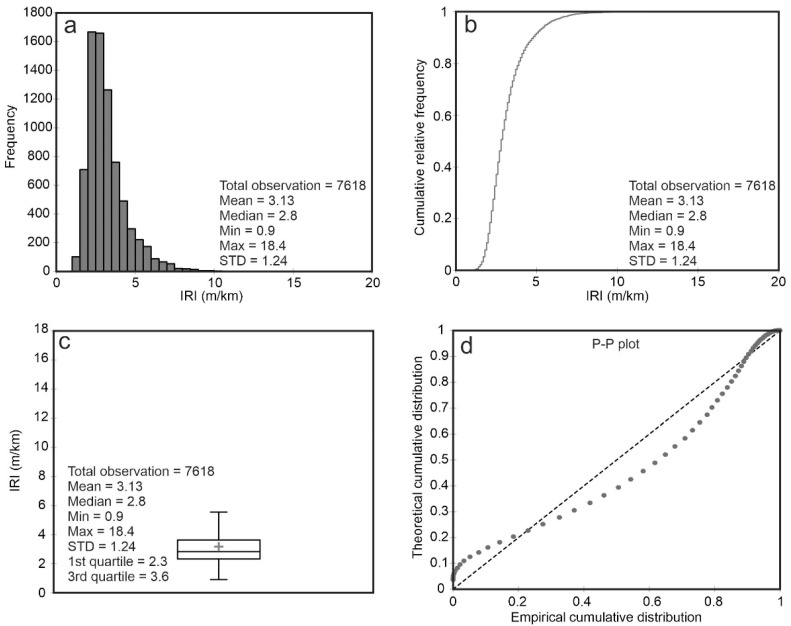
(**a**) Histogram of IRI measurements for the study area. (**b**) Empirical cumulative distribution of IRI measurements. (**c**) Boxplot of IRI measurements. The “+” sign indicates the mean IRI value. (**d**) Probability–probability (P-P) plot of the empirical cumulative distribution versus the theoretical cumulative distribution.

According to Sayers and Gillespie (1986), roads with IRI values between 1 and 4 m/km denote new pavement [5]. Figure 3a,b show that the IRI was recorded at 7618 points. The minimum and maximum in situ IRI values are 0.9 m/km and 18.4 m/km, respectively. The mean and median of the measurements are 3.13 m/km and 2.8 m/km, respectively, which implies that the major roads across the province have good overall quality. The roads studied are well maintained except for several routes. Figure 3c illustrates the distribution of IRI measurements in nonsymmetrical regions, and the first and third quartiles are 2.3 m/km and 3/6 m/km, respectively. The probability–probability plot (P-P) shown in Figure 3d can be used to test the fitness of theoretical and empirical distributions and to depict the skewness of the IRI distribution, which is not quite normal here (positive skew).

### 2.2. Remote Sensing Data

Remote sensing optical data obtained from UAVs or airborne/spaceborne platforms are mainly used for the extraction of roads, as described in several previous studies [18,19,20,21,22]. Meanwhile, synthetic aperture radar (SAR) data, especially high-resolution X-band SAR datasets, focus on the quality of the road. In previous studies, such high-resolution X-band SAR datasets were compared with IRI data in the U.S. and Iran, where bad-quality roads appear brighter in the SAR images than good-quality roads because rough surfaces have stronger backscattering properties [3,4]. However, despite the rapid growth of remote sensing, many transportation engineers have insufficient experience to practically apply remotely sensed data for their demands. On the other hand, remote sensing experts are not familiar with the engineering demands within the transportation sector. The interpretation of SAR data is more complex than that of optical data due to the side-looking nature of the former and its inherent limitations, such as foreshortening. Here, we focus on high-resolution optical data from Sentinel-2A and B within the study area and conduct novel research to develop equations that explain the roughness of primary and secondary roads. As mentioned above, we measured over 1300 km of roads in East Azerbaijan Province of Iran. To cover all the measured areas, we obtained 6 Sentinel-2 images (100 km × 100 km) with the closest available timestamps to each day on which the in situ measurements were obtained, from 10 October 2020 to 25 October 2020. The Sentinel-2 imagery has 12-bit radiometric resolution, 10 m spatial resolution, and 13-band spectral resolution. The bottom-of-atmosphere (BOA) reflectance values in the images from the MultiSpectral Instrument (MSI) were extracted from the 1C products of Sentinel-2, which are not atmospherically corrected. The Sentinel-2 mission has two polar-orbiting satellites—namely, Sentinel-2A and Sentinel-2B. As shown in Figure 2b and Table 1, two Sentinel-2 images (acquired on 10 and 23 October 2020) were obtained from Sentinel-2A, while the remaining images (25 October 2020) were obtained from Sentinel-2B. The mosaicked image covers approximately 90,000 km^2^ of the study area. Except for a gap between images #1 and #3, the regions containing the IRI measurements are well covered in the mosaicked image. Once the radiometric calibration of the mosaicked image is performed, the optimum bands that contain the highest variance (i.e., information) can be selected using the optimum index factor (OIF). 

### 2.3. Framework

**Figure 4:**
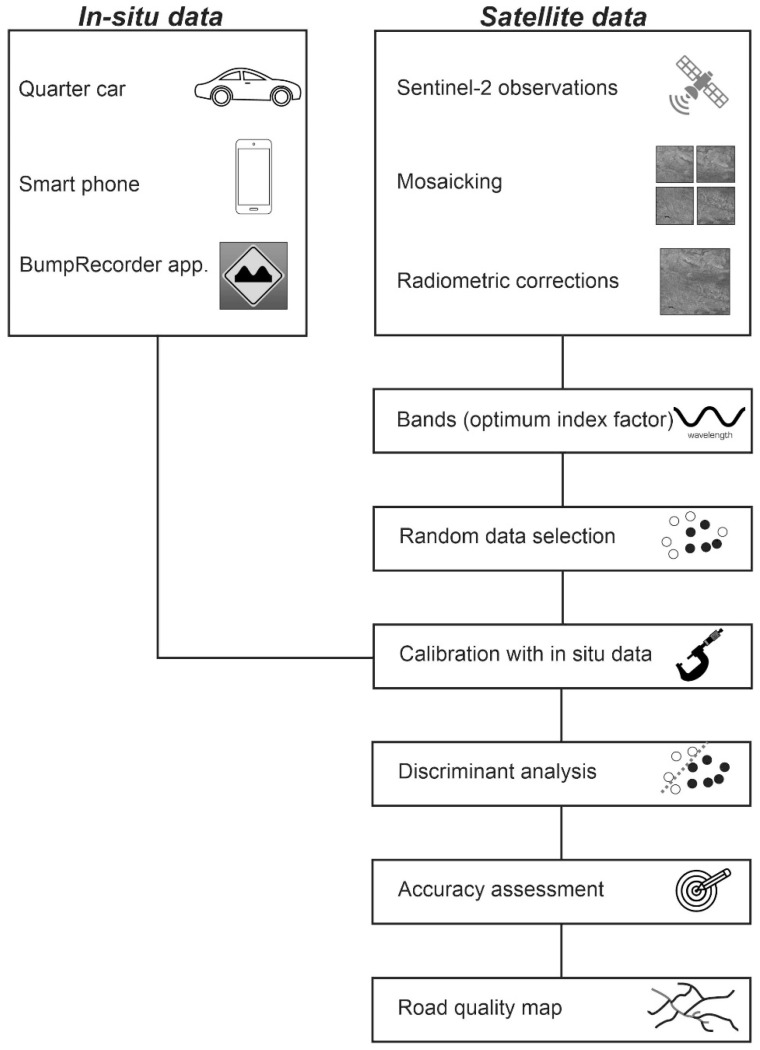
Workflow of road quality mapping based on in situ and satellite datasets.

The framework shown in Figure 4 shows the details of each step—namely, data calibration, discriminant analysis, and accuracy assessment.

#### 2.3.1. Optimum Index Factor 

The OIF is a measurement that is calculated for selecting the most ideal bands to be used for color composites or for preserving the maximum amount of information (highest standard deviation) in an image with minimal duplication [23,24,25]. The OIF calculation method starts with the creation of a map list that contains 13 Sentinel-2 bands. Once this list of multispectral bands is available, the variance–covariance matrix (correlation matrix) of the bands can be calculated according to the map list. To create any OIF, the map list should contain at least 3 raster images that are all georeferenced in the same domain. A simple OIF calculation method is as follows:

(1) $$\mathrm{OIF}=\;\frac{\sum_{k=1}^{3}s_{k}}{\sum_{j=1}^{3}Abs\left(r_{j}\right)}$$

where $s_{k}$ is the standard deviation for the corresponding band (k) and j is the absolute value of the correlation coefficient between two of the three bands in the calculation.

#### 2.3.2. Discriminant Analysis

In this study, the discriminant analysis was structured based on independent variables (i.e., spectral bands such as $b_{2}$, $b_{3}$, $b_{4}$, and $b_{8}$) and a binary map (the dependent variable). Note that for the binary map of the study area, “0” was assigned to good roads and “1” was assigned to bad roads. The binary map was produced from in situ IRI measurements conducted using a QC. Although these IRI measurements varied in the range between 0.9 m/km and 18.4 m/km, a simple assumption (e.g., good and bad roads) helped us to categorize the values into two qualitative groups: 0 for good roads and 1 for bad roads. Thus, a qualitative rather than a quantitative discriminant analysis was chosen to predict new scores based on the independent variables and the description of the binary map [26,27,28,29]. Here, discriminant analysis is a type of linear regression that seeks to find the relationship among several classes of objects. We regressed the independent variables for both the OIF and norm R results on the dependent variables and assigned a specific value for each sample point in the ground truth database. Table 2 shows the details of the linear regression for the independent and dependent variables. The discriminant score (Z) for the OIF and norm R can be defined as follows:

(2) $$Z_{OIF\_i}=\;a_{1}+(a_{2}\times b_{i2})+\left(a_{3}\times b_{i3}\right)+\left(a_{4}\times b_{i4}\right)+(a_{5}\times b_{i8})$$

(3) $$Z_{R\_i}=a_{1}+a_{2}\times N_{i}$$

Here, i represents the number of points on the IRI map. Because of the image gap (Figure 2b), the total number of points is reduced from 7618 to 7313. $a_{1}$ is the intercept value, and $a_{2}$, $a_{3}$, $a_{4}$, and $a_{5}$ are coefficients of the regression analysis for the blue, green, red, and near-infrared bands, respectively. $Z_{OIF\_i}$ and $Z_{R\_i}$ are the probability values for the OIF and norm R, respectively, which can be interpreted as the probabilities of being a “good” or a “bad” road. To define whether the calculated values are in the good or bad groups, we defined a cutoff value for the calculated discriminant scores as follows:

(4) $$C=\;\frac{(n_{0}\times {\bar{z}}_{0})+\left(n_{1}\times {\bar{z}}_{1}\right)}{\left(n_{0}+n_{1}\right)}$$

where C is the cutoff value, $n_{0}$ and $n_{1}$ are the numbers of good and bad roads, respectively, and ${\bar{z}}_{0}$ and ${\bar{z}}_{1}$ are the average values of the discriminant scores for good and bad roads, respectively. As shown in Table 2, the cutoff values of the OIF and norm R classifications are 0.211 and 0.207, respectively. This means that the roads with a lower value than the cutoff were sorted into group “0” and the roads with a higher value than the cutoff were sorted into group “1”. In other words, if a point from the database has a Z value smaller than the calculated C, the algorithm will categorize it as a “good” point (road), whereas if one point from the database has a Z value larger than the calculated C, the algorithm will categorize it as a “bad” point.

## 3. Results

**Figure 5:**
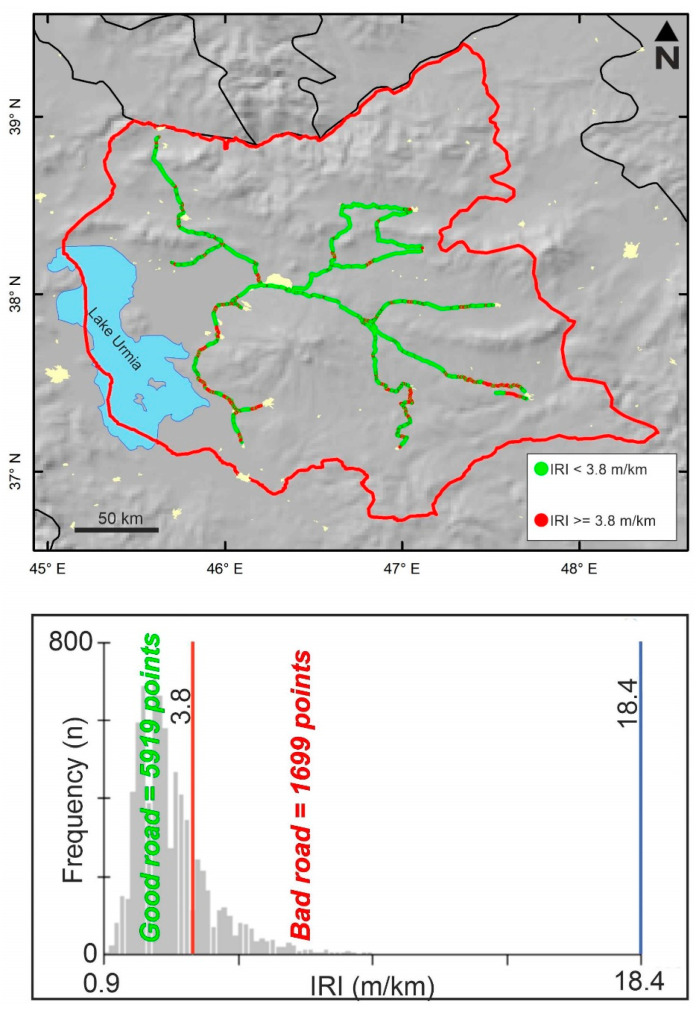
Binary road quality map based on a Jenks classification for IRI measurements.

Generally, we found an acceptable fit between the spectral information within the Sentinel-2 images and the in situ measurements, but the road quality map generated from the latter (graded colors in Figure 2b) was not helpful for discriminant analysis. To produce meaningful results for facile interpretation, the IRI measurements should be presented in a simpler format than the format traditionally used for these road quality maps. Due to the level of noise and the standard deviation of the measurements, as shown in Figure 5, all of the IRI measurements were categorized into two main classes: “good” and “bad” roads. Since road maintenance guides do not clearly define the metrics for classifying good and bad roads, we used the Jenks natural break optimization thresholding approach. This method is a data clustering technique for determining the most ideal arrangement of the IRI values into good and bad classes. Notably, Jenks optimization operates on a univariate classification, which maximizes the differences between the good and bad classes. Simply put, this method attempts to reduce the intraclass variance while maximizing the interclass variance. This goal is logical for our work because the IRI measurements can be divided into two categories where the difference in the IRI is relatively large. We adopted the following threshold for the classification of every measurement point:

(5) $${\mathrm{IRI}}_{\mathrm{threshold}}=\{\begin{array}{c} \mathrm{Good}\;\mathrm{road}:\mathrm{IRI}<3.8\;m/\mathrm{km}\\ \mathrm{Bad}\;\mathrm{road}:\mathrm{IRI}\geq 3.8\;m/\mathrm{km} \end{array}$$

**Figure 6:**
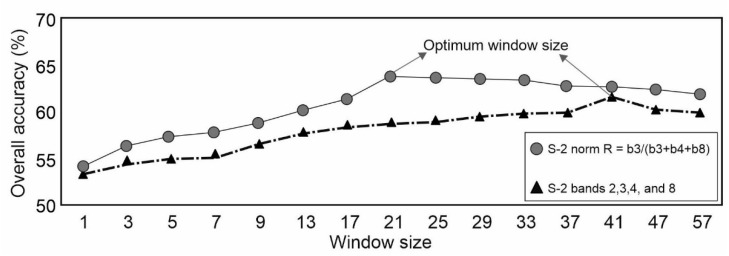
Overall accuracy of the OIF and norm R results using different window sizes.

**Figure 7:**
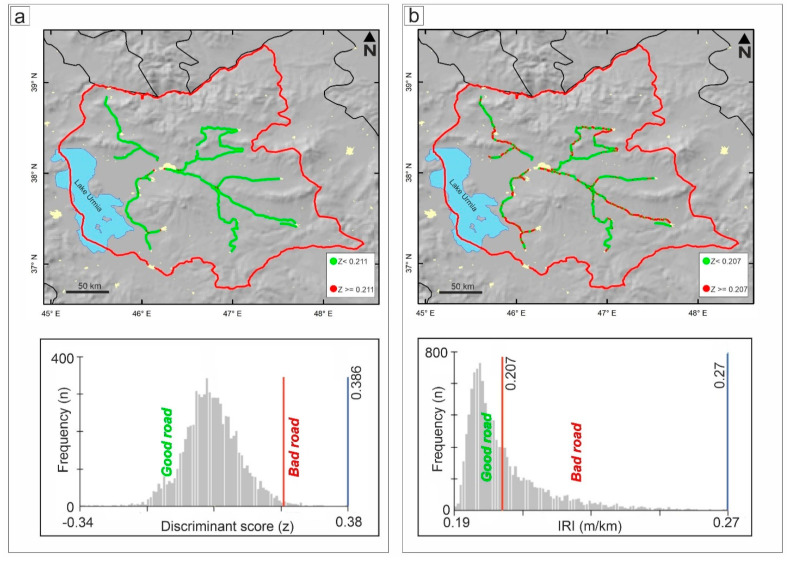
(**a**) Binary road quality proxy map (RQPM) deduced from the OIF and discriminant analysis. (**b**) Binary RQPM deduced from the norm R and discriminant analysis.

As we divided the IRI measurements into two groups (Figure 5), we also created a binary map using discriminant analysis which is an essential step for the analysis. Sentinel-2 images are composed of 13 bands at resolutions of 10 m, 20 m and 60 m. Based on the OIF, we selected useful bands that maintained the highest variance and ignored redundant information. These OIF results showed that the combination of bands 2, 3, 4 and 8 could provide the most information. Thus, discriminant analysis in different window sizes from 3 × 3 to 57 × 57 was carried out for these bands. The results show that among the 7618 IRI points, 5919 points denote good roads, and 1699 points signify bad roads (Figure 5). To apply the discriminant machine learning approach to the binary classification map, we employed a statistical measure—namely, the OIF—which combines only the useful multispectral bands in terms of the explained variance and standard deviation [23,24]. The OIF was utilized to reduce the complexity of the multispectral Sentinel-2 dataset and improve the discrimination accuracy for good and bad roads. The combination of the three bands with the largest OIF values as a red, green, blue (RGB) color composite yielded the most useful information while minimizing the amount of duplicated information. Here, we found that bands 2, 3, 4 and 8 had the most information; among them, the combination of bands 3, 4 and 8 yielded the largest OIF equal to 43.656. The second-largest OIF was produced by the combination of bands 2, 4 and 8 (OIF = 39.970). The discriminant analysis was conducted in different window sizes. Since the bidirectional reflectance distribution function and mix of pixels (MIXEL) for neighboring features (e.g., road and vegetation) can increase the amount of false spectral information during discriminant analysis, we applied different window sizes to maintain the real reflectance of the road surface [30,31]. The first model was based on the original data (1 × 1), and the other window sizes were 3 × 3, 5 × 5, 7 × 7, 9 × 9, 13 × 13, 17 × 17, 21 × 21, 25 × 25, 29 × 29, 33 × 33, 37 × 37, 41 × 41, 47 × 47, and 57 × 57. As shown in Figure 6, for bands 2, 3, 4 and 8, the most ideal window size for road classification was 41 × 41, and the corresponding discriminant equation is as follows:

(6) $$Z_{i}=\;0.317+0.00138b_{2}-0.00149b_{3}+0.0002b_{4}+0.000087b_{8}$$

where $b_{2}$, $b_{3}$, $b_{4}$, and $b_{8}$ are Sentinel-2 bands 2, 3, 4 and 8, respectively. The overall accuracy (OA) of the abovementioned technique for a 41 × 41 window is 61.0% (kappa ~0.51), but the classification of bad roads is still poor (accuracy of ~19%). Figure 7a indicates that the roads were classified according to a normal distribution. Since the total number of points (7313) was less than the number of in situ points (due to the gap in the mosaicked Sentinel-2 image), 7162 points were identified as good roads (green), while 151 points were identified as bad roads (red). The cutoff point was 0.211: values beyond this threshold were grouped into the bad road category. Details of the categorization based on this cutoff value and the corresponding accuracies are given in Table 3.

As an alternative, we applied another index (norm R) introduced by https://www.indexdatabase.de (access date 15 December 2020). Here, the norm R index was selected based on empirical tests of replacement of the bands. This index was suitable for our purposes since it is simpler than the combination of OIF bands and, like the OIF, it contains the maximum amount of information because the red, green, and NIR (near-infrared) bands all contribute to this index. The norm R is defined as follows:

(7) $$N=\;\frac{b_{3}}{b_{8}+b_{4}+b_{3}}$$

where N is the norm R and $b_{3}$, $b_{4}$ and $b_{8}$ are the green, red and near-infrared bands of Sentinel-2, respectively. Figure 6 shows the OA for different window sizes. Equation (8) is the discriminant equation for the optimum window size (21 × 21) produced from the norm R index:

(8) $$Z_{R}=\;0.342-0.425\times N$$

**Figure 8:**
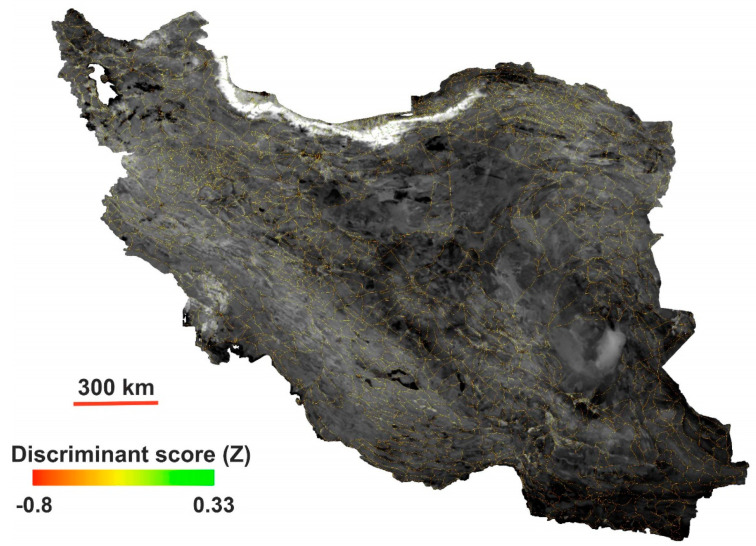
Nationwide RQPM deduced from the discriminant model of norm R.

Figure 7b indicates that, using this index, the roads are classified with a skewed distribution. Based on the estimated cutoff point (0.207), among the 7313 points, 4641 points were identified as good roads, while 2672 points were identified as bad roads. The OA for linear discriminant analysis on a window size of 21 × 21 was approximately 65% (kappa ~0.59), and the classification accuracy of bad roads was improved to 40%. The details of this categorization based on the corresponding cutoff value and its accuracies are given in Table 3. Equations (6) and (8) permit the calculation of road quality proxy maps (RQPMs) for large-scale regions on the basis of the existing raster pixel values and regressed dataset. Figure 8 shows the nationwide RQPM of the territory of Iran produced from Equation (8) and the mosaic of 23 Sentinel-2 images in the Google Earth Engine (GEE) for bands 2, 3, 4 and 8 during October 2020.

## 4. Discussion

Discovering local damages or local road unevenness using Sentinel-2 dataset (10 m spatial resolution) is challenging. Additionally, in dense urban areas surrounded by tall buildings and trees, IRI measurements could be biased (due to weak GPS signals, traffic jams, etc.). Thus, the field data gathered from highways and first-degree roads (every 200 m) together with Sentinel-2 spectral information can be used for road quality mapping over large-scale areas. Further investigations using very high resolution (VHR) images from commercial satellite images (e.g., WorldView-2, Spot, etc.) might help to discover different aspects of road quality mapping from space. The developed discriminant models (for OIF and norm R) in Equations (6) and (8) permit the calculation of RQPMs over large-scale areas based on the existing correlation between road roughness and raster pixel values, thereby reducing the hazard mitigation costs and time-consuming task of in situ road quality mapping. Despite its advantages, such as its compatibility with multiple dependent variables, reduced error rate, and simpler interpretation between grouped values, there are still some disadvantages to discriminant analysis. One such disadvantage is that discriminant analysis assumes the relationship between variables is linear although the relationship is not exclusively linear, especially for the backscattering coefficient values extracted from X-band SAR images. In particular, the relationship between the IRI and backscattering coefficient is not linear for whole roads (e.g., interstate and secondary roads) because the IRI is measured indirectly, and the relationship between the IRI and pavement roughness is somewhat complicated [3,32]. As mentioned before, the classification accuracy for bad roads is low, which is probably related to the inherent disadvantage of discriminant analysis being sensitive to outliers. Hence, an unbalanced number of points in the good and bad categories could have caused the poor segregation results. In the future, it will be important to create more robust models, increase the number of samples in difficult areas (e.g., with bad roads), and apply post-processing techniques such as generative learning or new machine learning algorithms compatible with any type of dataset.

It must be noted that tall or dense vegetation can negatively affect the results of road quality mapping. Since the study area has semi-arid climate, the vegetation was not a challenging problem. But to reduce the effects of vegetation, the mosaicked Sentinel-2 map was also masked using 10 m buffering and OSM. Other techniques such as principal component analysis (PCA) might be helpful to discover different aspects of road mapping, but the in situ IRI is a physical parameter that cannot fully be explained by pixel values. In addition, for a nationwide road quality analysis using GEE, calculation of PCA will be more difficult due to limited cloud storage.

## 5. Conclusions

We conclude that while the transportation sector is growing due to many reasons, such as socioeconomic demands, remote sensing application techniques should not only be focused on routine change detection issues but also on monitoring the quality of the features in detail. This study proposed a qualitative remote sensing approach for the segregation of good and bad roads based on IRI measurements and spectral information. We analyzed 4 bands of 6 multispectral Sentinel-2 images over East Azerbaijan Province in Iran and developed linear models to describe the correlation between the spectral band information and IRI data. These models can be used for other case studies on a nationwide scale; however, it must be noted that the IRI measurements were gathered under clear weather conditions between 10 °C and 30 °C with a long chassis car (CHANGAN-CS35). The altitude of the study area is approximately 1500 m above mean sea level (MSL), and the average speed of the QC was approximately 80 km/h. Accordingly, the limited transferability of the developed functions to other areas may reduce the road quality mapping capability elsewhere. In other words, for different areas or states, the development of domestic functions compatible with geographical specifications and road construction regulations is highly recommended. 

## Figures and Tables

**Table 1:** Detailed information about the Sentinel-2 images used for this study. The MSI2A instrument provides bottom-of-atmosphere (BOA) reflectance values for the Sentinel-2 images.

ID (#)	Sensor	Product Type	Date (yyyy/mm/dd)	Area (km^2^)
1	Sentinel-2A	MSI2A	2020/10/10	10,000
2	Sentinel-2A	MSI2A	2020/10/23	10,000
3	Sentinel-2B	MSI2A	2020/10/25	10,000
4	Sentinel-2B	MSI2A	2020/10/25	10,000
5	Sentinel-2B	MSI2A	2020/10/25	10,000
6	Sentinel-2B	MSI2A	2020/10/25	10,000

**Table 2:** Statistics for the linear regression analysis of the optimum index factor (OIF) and norm R dependent and independent variables.

OIF (41 × 41)		Norm R (21 × 21)	
Number of points	7313	Number of points	7313
R-squared	0.026	R-squared	0.0007
Multiple R	0.163	Multiple R	0.0275
Standard error	0.4	Standard error	0.405
$a_{1}$ (intercept)	0.317	$a_{1}$ (intercept)	0.342
$a_{2}$	0.00138	$a_{2}$	−0.425
$a_{3}$	−0.00149	$a_{3}$	-
$a_{4}$	0.0002	$a_{4}$	-
$a_{5}$	0.000087	$a_{5}$	-
Cutoff score	0.211	Cutoff score	0.207

**Table 3:** Road classification accuracy based on the discriminant scores ($Z_{i}$ and $Z_{R}$ ), optimum index factor (OIF) and norm R.

Sentinel-2 Classification (OIF)		Sentinel-2 Classification (Norm R)	
$Z_{i}>0.211$	7162	$Z_{R}>0.207$	4641
$Z_{i}\leq 0.211$	151	$Z_{R}\leq 0.207$	2672
Misclassified roads	2852	Misclassified roads	2560
Total correct roads	4461	Total correct roads	4753
Total accuracy	61%	Total accuracy	65%
